# Three Cases of Extirpation of the Eye

**Published:** 1861-12

**Authors:** E. L. Holmes

**Affiliations:** Chicago, Surgeon to the Chicago Charitable Eye and Ear Infirmlry


### ARTICLE III.

### THREE CASES OF EXTIRPATION OF THE EYE.

Reported to The CHICAGO MEDICAL SOCIETY

By E. L. HOLMES, M. D., of Chicago,

SURGEON TO THE CHICAGO CHARITABLE EYE AND EAR INFIRMLRY.

If it is in order, Mr. President, I will call the attention of the Society to three pathological specimens of the eye, which I have had occasion to extirpate for three different forms of disease. The specimens are preserved in a saturated solution of bichromate of potash, which has the property, not only of preserving the tissues, but also of coagulating the vitreous humor so firmly, that it retains its natural form, when its coverings are removed.

The largest specimen is from a young woman, living in the country, who had been affected four years with hydrophthalmos. The eye had gradually become so enlarged, that the lids could be no longer closed ; a large staphyloma of the sclerotic was forming upon the upper and inner portion of the globe, presenting the characteristic dark blue appearance, caused by the great attenuation of this membrane: the cornea was nearly twice its natural size, and exceedingly thin, as if it had been stretched to its utmost capacity by the pressure of the fluids within; the patient was already suffering from irritation and deep-seated pain in the eye; sight had long been hopelessly lost.

Surgical interference was there imperative. The staphyloma of the sclerotic had so far progressed, that any attempt to reduce the size of the eye by simple puncture, or even by removing the cornea would possibly, if not probably, be followed by a reproduction of the staphyloma, as is not unfrequently the case in such an affection. The eye was therefore extirpated in the manner which I will soon describe.

The wound healed in a few days, and at the end of a month, the patient returned to the city for an artificial eye, which was worn without the least difficulty, and fortunately, moved much more freely than usual in such cases.

The second specimen is an eye which I removed from an Irishman, connected with the Irish Brigade, of this city. In May last, he consulted me regarding his eye, which he stated, had been injured three weeks before, by a severe blow. On examination, I found the globe atrophied, vision wholly extinct, the iris and cornea united and much changed in structure. There was a large cicatrix on the upper portion of the globe a few lines from the cornea, the result, evidently, of a rupture of the sclerotic. The patient complained of severe and constant pain in and around the eye. There was great tenderness on pressure in the region of the ciliary processes.

As it is seldom I have found any constitutional treatment beneficial in such injuries, I advised the immediate removal of the eye, not only to relieve present pain, but also to preserve the other eye from sympathetic inflammation. The patient, however, would not listen to this advice. Two months after this, he again visited me, stating that he had constantly suffered severe pain, which had become worse at night, and that he was willing to submit to any operation I thought best. His general health was also somewhat impaired. The eye presented nearly the same appearance as when I first saw it. Five days after the removal of the eye, the patient left Chicago with his brigade, wholly free from pain, with health improving.

The history of the third case is not without interest, as being an example of the great difficulty in relieving irido-choroiditis by medical treatment, when the pupil has become filled with organized lymph, and attached to the lens. The patient, aged nineteen years, stated that he had been subject to constant pain, with violent exacerbations for five years, following a severe inflammation, which, I suppose, from his description and the appearance of the eye, to have been irido-choroiditis. He stated that he had been treated, for a long time, without success, by one of the most skilful physicians of Glasgow, Scotland. The eye was atrophied, the pupil being closed with organized lymph, and the iris much discolored. The removal of the eye gave immediate relief. The patient suffered less inconvenience from the effects of the operation than before, and even felt better able to labor the next day, than for a long time previous.

The operation as recommended by Critchett and others, is very simple. The eye being firmly secured by means of a hook or toothed forceps, an incision is made parallel with the circumference of the cornea, through the conjunctiva. Each of the recti muscles is then seized by a blunt hook, passed beneath it, and divided as in the operation for strabismus. The eye at once protrudes, when a pair of blunt scissors (curved), can be passed behind the globe, and the nerve severed, close to its entrance through the sclerotica. The section of the oblique muscles completes the operation.

There is usually very little loss of blood. Still, it is well, I think, to prevent the formation of large coagula in the orbit, if possible, by injecting cold water freely after the operation. The only dressing necessary, is a simple wet compress. The lids are liable to become slightly œdematous, but there is little pain or inconvenience, so little in fact, that patients often persist in sitting up the next day, or even in going out. The wound is generally completely healed at the end of a fortnight.

The disadvantages of this operation consist almost wholly in the fact, that there is a smaller "stump" for an artificial eye, which is apt to have less motion, than when the anterior portion of the eye alone is removed And yet, the muscles, the conjunctiva and the lymph, which is thrown out in quite large quantities, and which sometimes becomes organized, often form a much better support for an artificial eye, and give it more motion, than one might suppose.

In the vast majority of simple cases of staphyloma corneæ, and of hydrophthalmos in its early stages, the "amputation" of the cornea and iris, will terminate favorably. Occasionally, however, the wound does not heal readily, the remainder of the eye becomes inflamed, which necessitates its removal. Occasionally, also, severe hemorrhage from rupture of the vessels of the choroid and retina produce large coagula behind the vitreous humor, causing excessive inflammation and suppuration.

In cases like those just reported, especially the last two, and in cases where foreign bodies, or a dislocated lens have been retained some time in the eye, causing chronic inflammation of the whole eye, we believe its entire removal will not only give less trouble to the patient, as regards the diseased eye, but will also, with more certainty, prevent sympathetic inflammation in the other one.

---

Cases of lead poisoning from taking snuff containing lead, are reported in the English journals.

